# Regulation of oxytocin receptor gene expression in obsessive–compulsive disorder: a possible role for the microbiota-host epigenetic axis

**DOI:** 10.1186/s13148-022-01264-0

**Published:** 2022-03-31

**Authors:** Claudio D’Addario, Mariangela Pucci, Fabio Bellia, Antonio Girella, Annalaura Sabatucci, Federico Fanti, Matteo Vismara, Beatrice Benatti, Luca Ferrara, Federica Fasciana, Laura Celebre, Caterina Viganò, Luca Elli, Manuel Sergi, Mauro Maccarrone, Valeria Buzzelli, Viviana Trezza, Bernardo Dell’Osso

**Affiliations:** 1 Faculty of Bioscience, University of Teramo, Teramo, Italy; 2 Department of Clinical Neuroscience, Karolinska Institutet, Stockholm, Sweden; 3 Department of Mental Health, Department of Biomedical and Clinical Sciences “Luigi Sacco”, University of Milano, Milano, Italy; 4 Department of Biotechnological and Applied Clinical Sciences, University of L’Aquila, L’Aquila, Italy; 5 European Center for Brain Research/Santa Lucia Foundation IRCCS, Rome, Italy; 6 Department of Science, Roma Tre University, Rome, Italy; 7 Faculty of Bioscience and Technology for Food, Agriculture and Environment, University of Teramo, Via Renato Balzarini, 1, 64100 Teramo, Italy; 8 Department of Psychiatry, Department of Biomedical and Clinical Sciences “Luigi Sacco”, Psychiatry Unit 2, ASST Sacco-Fatebenefratelli, Via G.B. Grassi, 74, 20157 Milan, Italy

**Keywords:** Oxytocin receptor, DNA methylation, Gene expression, Obsessive–compulsive disorder, Microbiota, Saliva

## Abstract

**Background:**

Obsessive–compulsive disorder (OCD) is a prevalent and severe clinical condition. Robust evidence suggests a gene-environment interplay in its etiopathogenesis, yet the underlying molecular clues remain only partially understood. In order to further deepen our understanding of OCD, it is essential to ascertain how genes interact with environmental risk factors, a cross-talk that is thought to be mediated by epigenetic mechanisms. The human microbiota may be a key player, because bacterial metabolites can act as epigenetic modulators. We analyzed, in the blood and saliva of OCD subjects and healthy controls, the transcriptional regulation of the oxytocin receptor gene and, in saliva, also the different levels of major phyla. We also investigated the same molecular mechanisms in specific brain regions of socially isolated rats showing stereotyped behaviors reminiscent of OCD as well as short chain fatty acid levels in the feces of rats.

**Results:**

Higher levels of oxytocin receptor gene DNA methylation, inversely correlated with gene expression, were observed in the blood as well as saliva of OCD subjects when compared to controls. Moreover, *Actinobacteria* also resulted higher in OCD and directly correlated with oxytocin receptor gene epigenetic alterations. The same pattern of changes was present in the prefrontal cortex of socially-isolated rats, where also altered levels of fecal butyrate were observed at the beginning of the isolation procedure.

**Conclusions:**

This is the first demonstration of an interplay between microbiota modulation and epigenetic regulation of gene expression in OCD, opening new avenues for the understanding of disease trajectories and for the development of new therapeutic strategies.

**Supplementary Information:**

The online version contains supplementary material available at 10.1186/s13148-022-01264-0.

## Background

Obsessive–compulsive disorder (OCD) is a psychiatric condition responsible for a significant impairment of daily functioning and reduction in quality of life, for both patients and their caregivers [1–3].

Available treatments for OCD are, as yet partially effective, psychotherapy and medications [4]. Of note, only one-third of people seek help for their OCD, and less than 10% receive evidence-based treatment [5, 6]. Moreover, OCD patients might present themselves in a relatively healthy way when compared to subjects with other psychiatric conditions, and diagnostic criteria might not be effective for subjects with subthreshold symptoms [7]. Additional research is thus urgently needed to elucidate the pathophysiology and the neurobiological bases of OCD. As yet, many studies have focused on the inherited component of OCD. However, the available data identifying DNA sequence-based causes are inconclusive with sometimes conflicting results [8]. Environmental risk factors (e.g., stressful life events, trauma and impoverished social context), known to be relevant in the development of OCD [9–11], can modify genes transcription regulation via epigenetic mechanisms [12]. How epigenetics might play a role in OCD has been already investigated in previous clinical studies comparing subjects with the disorder and healthy controls. For instance, Yue et al. [13] found that 2190 genes were differentially methylated in OCD. In particular, differential DNA methylation has been reported for gamma-aminobutyric acid type B receptor subunit 1 (GABBR1) in blood samples at birth and for myelin oligodendrocyte glycoprotein (MOG) and brain-derived neurotrophic factor (BDNF) genes at the time of diagnosis [14]. Also, lower DNA methylation and higher DNA hydroxymethylation have been reported in OCD patients [15]. Finally, lower methylation levels were observed at oxytocin receptor (*OXTR*) exon 2 [16], whereas higher levels have been reported at exon 3 [17, 18] in OCD. All the above-mentioned studies were performed on blood samples. Interestingly, a few studies have reported changes in genes regulation in saliva, where significantly higher DNA methylation levels were observed in the first intron of the SLC6A4 (serotonin transporter) gene in pediatric OCD patients compared to healthy controls and adult OCD patients [19]. Again, the *OXTR* gene was found to be epigenetically modulated, but in this case at the level of the first intron [20].

Epigenetic events in the cells are driven by endogenous metabolites responsible for the activity of all those enzymes needed for dynamic epigenetic modifications [21]. In the last few years, research started to investigate how gut microbiota metabolism may regulate the concentration and/or activity of metabolites in the host that lead to altered epigenetic marks [22–24]. The possibility of a microbiota-host epigenetic axis is thus attracting growing attention [25]. Such an axis needs to be interrogated also in mental illnesses, where the role of the gut microbiome appears particularly relevant [26, 27]. This is particularly true for OCD, where an impact of gut bacteria has been suggested [28–30].

Against this background, here we decided to further interrogate the epigenetic regulation of *OXTR* in OCD by analyzing its transcriptional regulation in both blood and saliva patients’ samples, evaluating in the latter also the distribution of the major bacterial phyla, compared to healthy subjects. In particular, we ascertained the role of gene DNA methylation and of oral microbiota in driving a potentially different modulation due to the disorder. It should be recalled that recent research is focusing on saliva samples as a valuable alternative to blood for molecular assays, also because some salivary components derive indeed from blood [31, 32]. Moreover, saliva collection is clearly less invasive and without the risks potentially occurring when using blood [33]. Remarkably, good quality DNA can be easily isolated from saliva and used not only for genetic studies [34] but also for epigenetic analysis [35–39]. Finally, salivary microbiota is stable enough for analysis of its composition, partly overlaps with gut microbiota and is affected by diet and lifestyle [40, 41]. Unsurprisingly, studies are now pointing out on the link between microbiota of mouth and gut of humans, and an “oral–gut axis” has been recently proposed [42]. In order to corroborate human data, we also analyzed molecular outcomes in socially isolated rats displaying, during a critical developmental window, behavioral abnormalities that might be evocative of certain behavioral symptoms are shown by OCD patients. We thus explored the transcriptional regulation of *OXTR* gene at central level (in the amygdala, hypothalamus and prefrontal cortex) as well as fecal short chain fatty acid levels.

## Methods and materials

### Subjects

A total of 64 patients (30 women and 34 men; age: 38.03 ± 13.77) followed up at the OCD tertiary outpatient Clinic of the University Department of Psychiatry of Milan, Luigi Sacco Hospital, were included in the study. Diagnoses were assessed by the administration of a semi-structured interview based on DSM-5 criteria (SCID 5 research version, RV) [43]. In case of psychiatric comorbidity, OCD had to be the primary disorder and illness severity was measured through the Yale-Brown Obsessive–Compulsive Scale [44]. The presence of medical condition and/or drug abuse represented exclusion criteria. All patients were for at least one month on stable pharmacological treatment chosen according to international guidelines in the field [4]. A total of 51 age and sex-matched controls (31 women and 20 men; age: 37.22 ± 13.48) were volunteers without any psychiatric disorder, as determined by the nonpatient edition of the SCID and no positive family history for major psychiatric disorders in the first-degree relatives [45]. All subjects had given their written informed consent to participate in the study, which included the use of personal and clinical data as well as blood drawing for genotyping and methylation analysis. The study protocol had been previously approved by the local Ethics Committee. Demographic and clinical characteristics for the OCD subjects as well as psychotropics used are shown in Table 1.

**Table 1:** Socio-demographic and clinical features of OCD patients

**SOCIO-DEMOGRAPHIC FEATURES**	
Gender (m;f)	34;30
Age at recruitment (mean ± SD)	38 ± 13.8
Education: graduated (%)	28.1
Employment: employed (%)	57.8
Married (%)	32.8
**CLINICAL FEATURES**	
Age at OCD onset (years, mean ± SD)	22.6 ± 10.3
Onset < 18 y (%)	34.4
**Family history of psychiatric disorder (%)**	
None	40.6
Unipolar Depression Family History	37.5
Anxiety Disorders Family History	12.5
Bipolar Disorders Family History	6.2
OCD Family History	4.7
**Psychiatric comorbidity (%)**	
None	31.2
Unipolar depression	26.6
Bipolar disorders	10.9
Anxiety disorders	15.6
Tic + Tourette	10.9
Others	9.4
**Other clinic variables**	
Age at first treatment (years, mean ± SD)	25.5 ± 11
Duration of untreated illness (months, mean ± SD)	48.9 ± 75.4
Duration of illness (years, mean ± SD)	16 ± 10.7
**Psychometric variables**	
Y-BOCS score (mean ± SD)	20.8 ± 9
**Current treatment (%)**	
Antidepressants (in combination)	90.6
Antidepressants (monotherapy)	21.9
Mood stabilizers	14.1
Antipsychotics	42.2
Benzodiazepines	28.1
Drug-free	7.8

OCD, obsessive compulsive disorder; Age at recruitment, Age at OCD onset, Age at first treatment, Duration of Untreated Illness, Duration of Illness, Y-BOCS score are reported as mean ± Standard deviation (SD)

### Animal model of social isolation

Female Wistar rats (Charles River, Calco (Lecco) Italy), weighing 250 ± 15 g, were mated overnight. Food and water were available ad libitum. Pregnant rats were singly housed in Macrolon cages (40 (length) × 26(width) × 20 (height) cm), under controlled conditions (temperature 20–21 °C, 55–65% relative humidity and 12/12 h light cycle with lights on at 07:00 h). On postnatal day (PND) 1, the litters were culled to eight animals (six males and two females), to reduce the litter size-induced variability in the growth and development of pups during the postnatal period. The experiments were carried out on the male offspring. On PND 21, the pups were weaned and randomly housed either one per cage (ISO group) or three per cage (CTRL group). The sample size was based on our previous experiments and power analysis performed with the software G power. The experiments were performed in agreement with the Animals in Research: Reporting in vivo Experiments (ARRIVE) guidelines [46, 47], with the guidelines released by the Italian Ministry of Health (D.L. 26/14) and the European Community Directive 2010/63/EU.

**Fig. 1:**
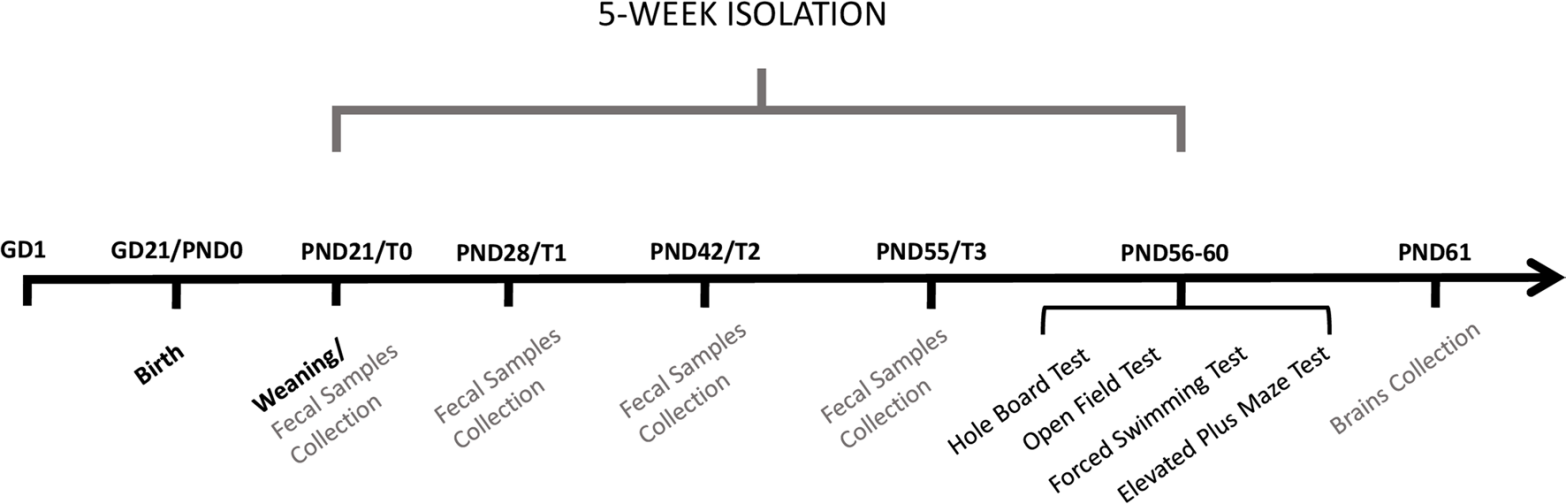
Schematic representation of the experimental design that was used to investigate the effects of social isolation in rats. Behavioral and molecular analysis was conducted at the indicated time points

After weaning at PND 21, ISO rats were housed individually for five weeks (from PND 21 to PND 54), while CTRL rats were housed in groups of three animals per cage for the same period (Fig. 1). Animals from both experimental groups performed behavioral tests at PNDs 55–58. At T0 (one day before isolation), T1 (one week after isolation), T2 (three weeks after isolation) and T3 (five weeks after isolation) fecal samples were collected and immediately stored at − 80 °C until use. At PND 61, rats were rapidly decapitated, their brains were removed and immediately frozen.

Details of the behavioral tests performed (Open Field, Hole-board, Elevated plus maze and Forced Swim) are described in Additional file 1.

### Molecular studies

**Fig. 2:**
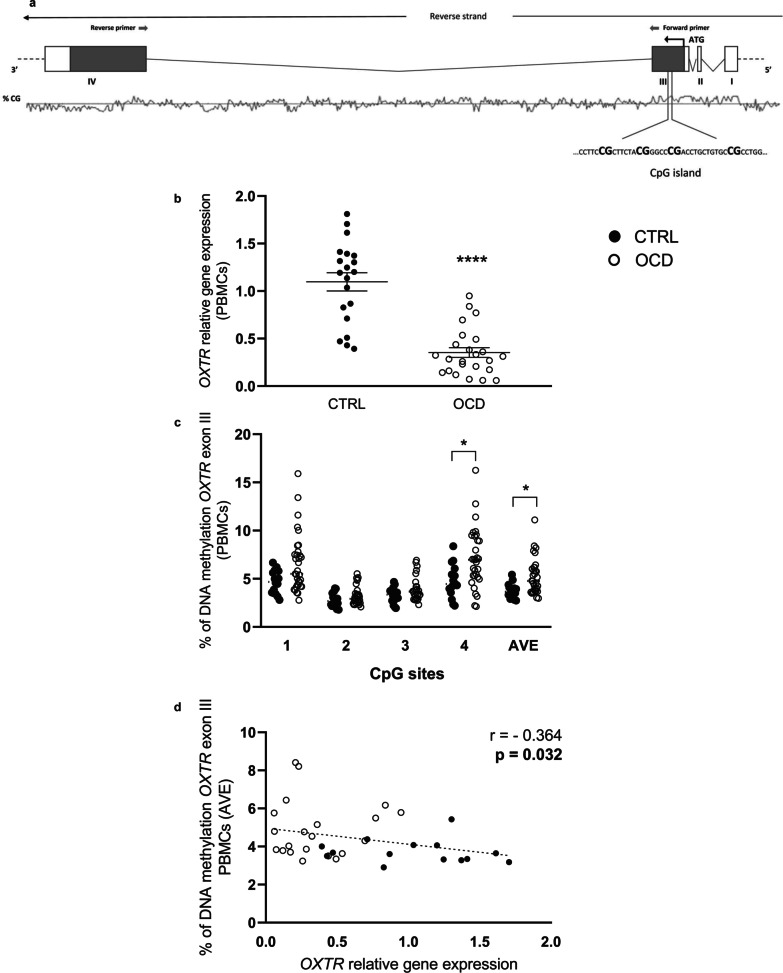
Transcriptional regulation of *OXTR* gene in PBMCs of OCD and control (CTRL) subjects. **a** Schematic representation of human *OXTR* gene. Translation start codon (ATG), exons and introns are depicted. Coding regions of exons are shown darker. Sequence of the CpG island under study is also reported. Bold text indicates the 4 CpG sites analyzed; **b** OXTR mRNA levels in PBMCs from patients diagnosed with OCD and CTRL subjects. Scattered plots represent 2 ^(−DDCt)^ values calculated by the DDCt method [****p < 0.001]; **c** *OXTR* exon III DNA methylation levels in PBMCs of OCD and CTRL subjects represented as scattered plot for individual CpG sites under study as well as of the average (AVE) of the 4 CpG sites [*p < 0.05]; **d** correlation between *OXTR* gene expression and % change of DNA methylation in the overall human population under study. Data were compared by Spearman's rank correlation coefficient

Nucleic acids were isolated from dissected rats brain regions (amygdala, hypothalamus and prefrontal cortex) and from human PBMCs, as previously described [48], to analyze mRNA abundance and DNA methylation levels of *OXTR* (see Figs. 2a and Additional file 1: Fig. S1 for analyzed sequences). Genomic DNA from saliva samples was prepared using a modified version of the salting-out method as described previously [49, 50]. Details for the analysis of genes expression and microbiota using human and rat genomic materials are reported in Additional file 1.

#### Analysis of DNA methylation

As previously described [15] 500 ng of DNA from each purified sample was subjected to bisulfite modification using the EZ DNA Methylation-GoldTM Kit (Zymo Research, Orange, CA, USA), inducing chemical conversion of unmethylated cytosine residues to uracil. The DNA methylation status of each CpG site in *OXTR* CpG island was assessed using a pyrosequencing assay. The schematic representation of the CpG islands under study at *OXTR* human and rat genes is shown in Fig. 2a and Additional file 1: Fig. S1, whereas the exact locations of the CpG sites analyzed are reported in Additional file 1: Table S2. DNA, after bisulfite treatment, was first amplified by PyroMark PCR Kit (Qiagen, Hilden, Germany) with a biotin labeled primer (Hs_OXTR_01_PM PyroMark CpG assay, PM00016821; Rn_Oxtr_02_PM PyroMark CpG assay, PM00546546) according to the manufacturer’s recommendations. PCR conditions were as follows: 95 °C for 15 min, followed by 45 cycles of 94 °C for 30 s, 57 °C for 30 s, 72 °C for 30 s and, finally, 72 °C for 10 min. Specificity of PCR products was then verified by electrophoresis. The sequencing was performed on a PyroMark Q24 ID using Pyro Mark Gold reagents (Qiagen, Hilden, Germany), after immobilizing the amplified product to Streptavidin Sepharose High-Performance (GE Healthcare, Chicago, IL, USA) beads via biotin affinity and denatured to allow the annealing with the sequencing primer. DNA methylation level was analyzed through the PyroMark Q24 ID version 1.0.9 software which calculates the methylation percentage mC/(mC + C) (mC = methylated cytosine, C = unmethylated cytosine) for each CpG site, allowing quantitative comparisons. The quantitative methylation results were expressed both as a percentage of every single CpG site and as the average of the methylation percentage of all the 4 CpG sites under study.

### Evaluation of short chain fatty acid levels

SCFAs extracted from rat fecal samples and samples derivatization for LC–MS/MS analysis were carried out following the method described by Han et al. [51] and Liebisch et al. [52] with slight modifications. Details are reported in Additional file 1.

### Statistical analysis

All results were expressed as mean ± standard error of the mean (SEM). Statistical differences in both human and animal samples were determined using GraphPad Prism® 8 (Graph-Pad Software, San Diego, CA). Data from the behavioral experiments in rats were analyzed with Student’s *t*-tests.

Changes in gene expression (*OXTR* mRNA levels and phyla quantification) and SCFA levels were analyzed using nonparametric Mann–Whitney test. DNA methylation at each CpG site was analyzed using the Mann–Whitney test and Sidak–Bonferroni correction was used for the multiple comparisons. All the data were compared by Spearman's rank correlation coefficient. The P-values < 0.05 were considered to be statistically significant.

## Results

### Human studies

#### Molecular analysis performed in PBMCs

We report a significant downregulation of *OXTR* mRNA levels in PBMCs of OCD subjects when compared to healthy controls (OCD: 0.35 ± 0.05; CTRL: 1.10 ± 0.10; *p* < 0.0001) (Fig. 2b) and, consistently, a significant increase in *OXTR* DNA methylation within gene exon III in OCD subject at CpG site 4 (OCD: 7.10 ± 0.56; CTRL: 4.75 ± 0.42; *p* = 0.036) and in the average of the 4 CpG sites investigated (OCD: 5.21 ± 0.32; CTRL: 3.74 ± 0.15; *p* = 0.006) (Fig. 2c). An inverse correlation between gene expression (2^(−DDCt)^ values) and DNA methylation considering the average of all the CpG sites analyzed was also observed (Spearman’s *r* = − 0.364, *p* = 0.032) (Fig. 2d). Moreover, stratifying data according to gender, we show a lower DNA methylation at CpG site 4 in PBMC of OCD men when compared to women (men: 5.65 ± 0.66; women: 8.47 ± 0.76; *p* = 0.045) (Additional file 1: Fig. S3a).

#### Molecular analysis performed in saliva

**Fig. 3:**
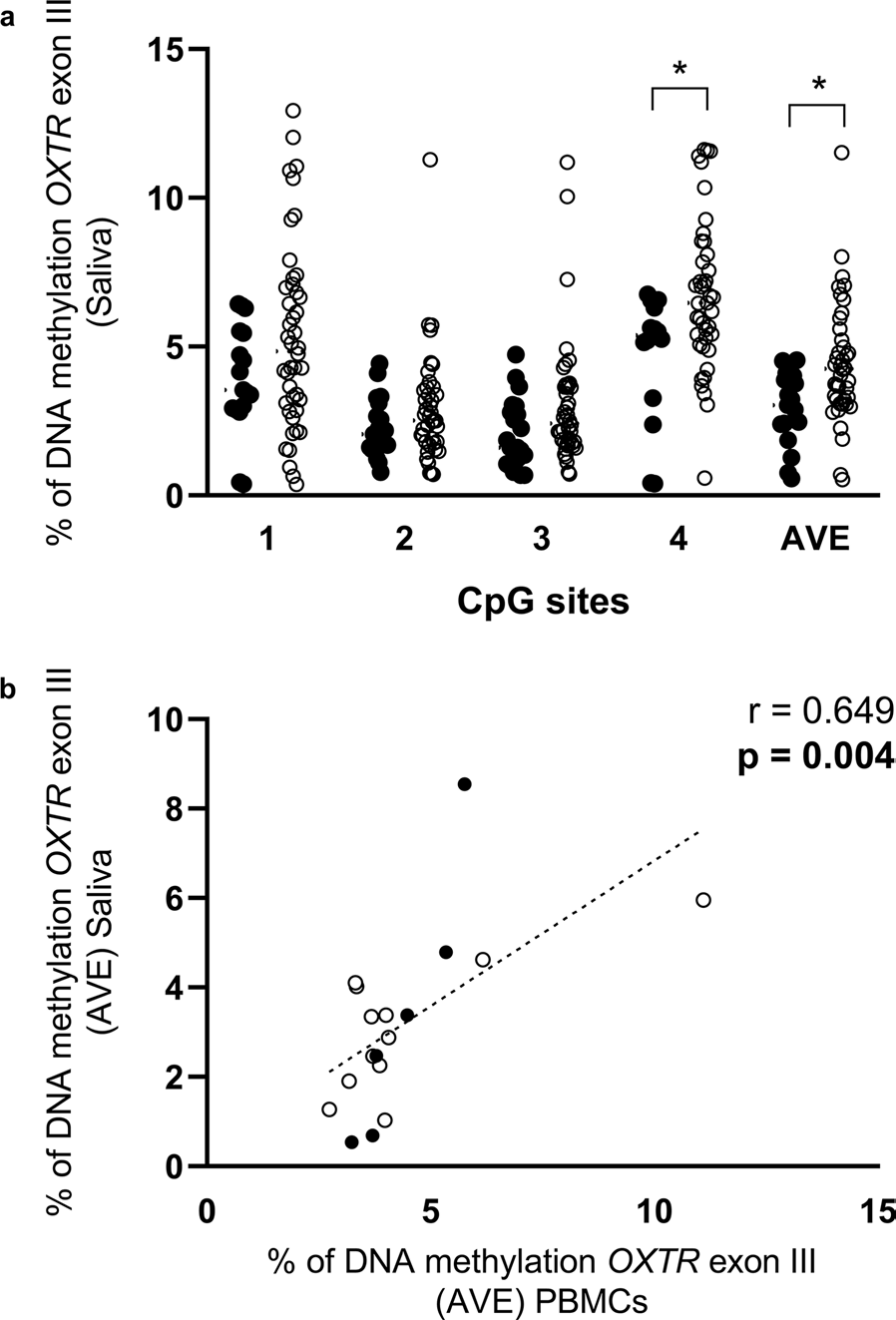
Transcriptional regulation of *OXTR* gene in saliva of OCD and control (CTRL) subjects. **a** DNA methylation levels of *OXTR* exon III in human saliva samples of OCD and CTRL subjects represented as scattered plot for individual CpG sites under study as well as of the average (AVE) of the 4 CpG sites [*p < 0.05]; **b** Spearman correlation analysis between AVE of DNA methylation from PBMCs (*x*-axis) and from saliva (*y*-axis)

A significant increase of *OXTR* DNA methylation levels in OCD patients compared to healthy subjects was observed again both at CpG site 4 (OCD: 6.76 ± 0.40; CTRL: 4.64 ± 0.58; *p* = 0.037) and in the average of the 4 CpG sites investigated (OCD:4.43 ± 0.31; CTRL: 2.95 ± 0.27; *p* = 0.021) (Fig. 3a). A significant positive correlation between the percentage of DNA methylation levels in PBMCs and in saliva, considering the AVE of the 4 CpG sites (Spearman correlation analysis: *r* = 0.649; *p* = 0.004) (Fig. 3b), was observed. In OCD women, levels in DNA methylation in the average of the 4 CpG sites were higher compared with respective matching healthy controls (OCD: 4.58 ± 0.35; CTRL: 3.33 ± 0.27; *p* = 0.049) (Additional file 1: Fig S3e). Moreover, the epigenetic mark significantly directly correlates with years from disease onset (*r* = 0.298; *p* = 0.043) (Additional file 1: Fig. S3g) and age in OCD patients (*r* = 0.338 , *p* = 0.029) but not in healthy controls (Additional file 1: Table S5). Finally, DNA methylation does not result altered when comparing subjects under any pharmacological treatment with drug-naïve patients (Additional file 1: Fig. S4) and no correlation was observed between *OXTR* DNA methylation and Y-BOCS values (Additional file 1: Table S6).

#### Microbiota characterization in saliva

**Fig. 4:**
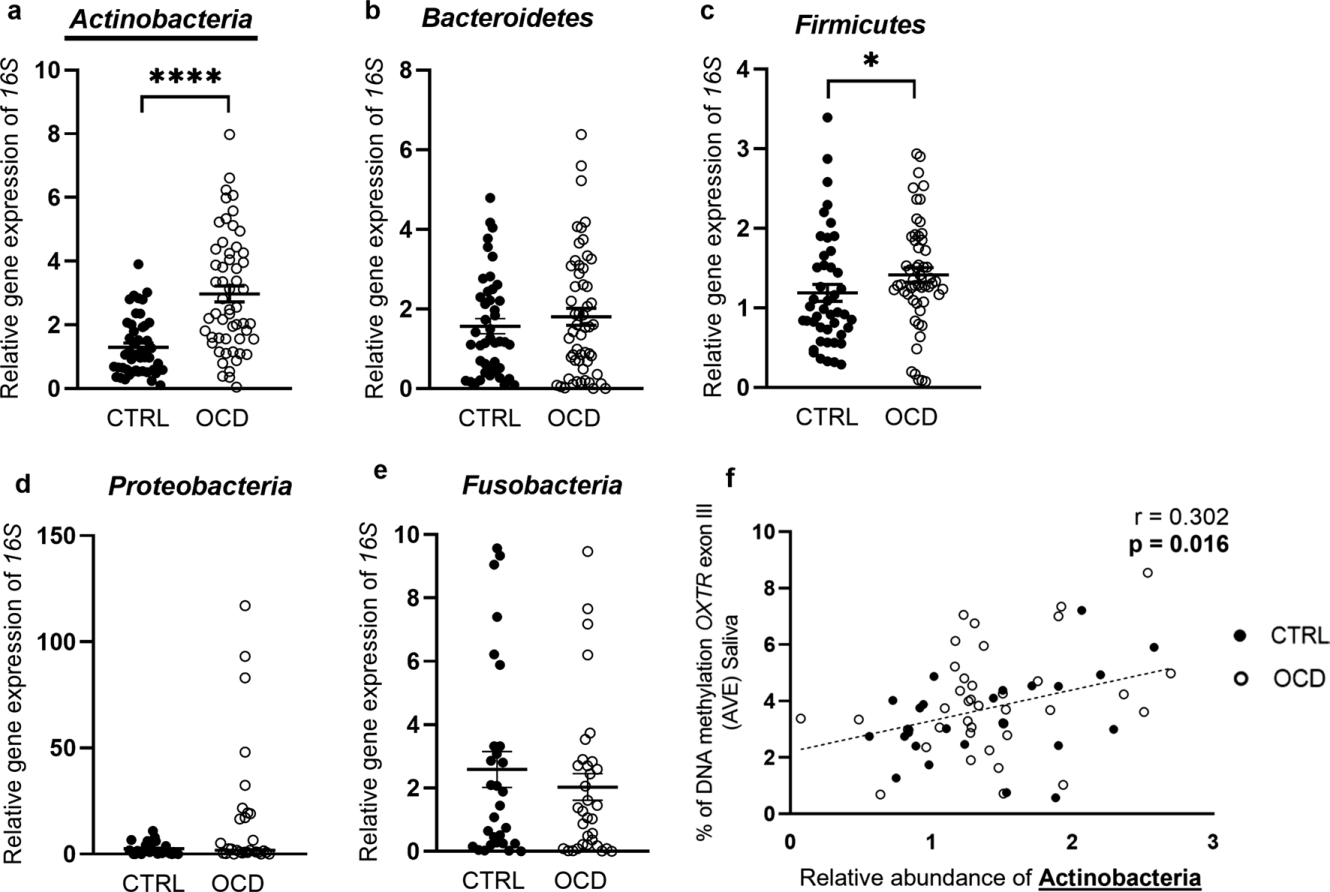
Relative abundance of the bacterial phyla (**a** Actinobacteria, **b** Bacteroidetes, **c** Firmicutes, **d** Proteobacteria, **e** Fusobacteria) quantified in the saliva of OCD and CTRL groups. Scattered plots represent 2^(−DDCt)^ values; **f** Spearman correlation analysis between DNA methylation AVE levels of the 4 CpG sites under study in saliva (y-axis) and relative abundance of Actinobacteria (*x*-axis). ****p < 0.0001, *p < 0.05 vs CTRL

The quantification of major phyla using rRNA-gene-based PCR is reported in Fig. 4a–e. A selective increase in *Actinobacteria* (CTRL: 1.30 ± 0.13; OCD: 2.97 ± 0.25; *p* < 0.0001, Mann–Whitney test) and *Firmicutes* (CTRL: 1.19 ± 0.11; OCD: 1.41 ± 0.09; *p* = 0.026) was observed in OCD subjects when compared to healthy individuals. No differences were observed in *Bacteroidetes* (CTRL: 1.57 ± 0.19; OCD: 1.81 ± 0.21; *p* = 0.706), *Proteobacteria* (CTRL: 2.56 ± 0.58; OCD: 17.13 ± 5.66; *p* = 0.079) and *Fusobacteria* (CTRL: 2.58 ± 0.56; OCD: 2.03 ± 0.42; *p* = 0.563). Moreover, it was observed a significant decrease in *Fusobacteria* to *Actinobacteria* ratio in OCD patients compared to controls (CTRL: 2.90 ± 0.72; OCD: 0.86 ± 0.44; *p* = 0.025. Figure not shown). No differences have been observed in *Firmicutes* to *Bacteroidetes* ratio in OCD and controls (CTRL: 2.16 ± 0.46; OCD: 2.13 ± 0.48; *p* = 0.902. Figure not shown). We also observed a significant correlation between the levels of DNA methylation (AVE) and the abundance of *Actinobacteria* (expressed as 2^(-DDCt)^ value) (*r* = 0.302, *p* = 0.016) (Fig. 4f), but not between the epigenetic mark and *Firmicutes* levels (*r* = 0.085, *p* = 0.498).

### Rat studies

#### Open field test

**Fig. 5:**
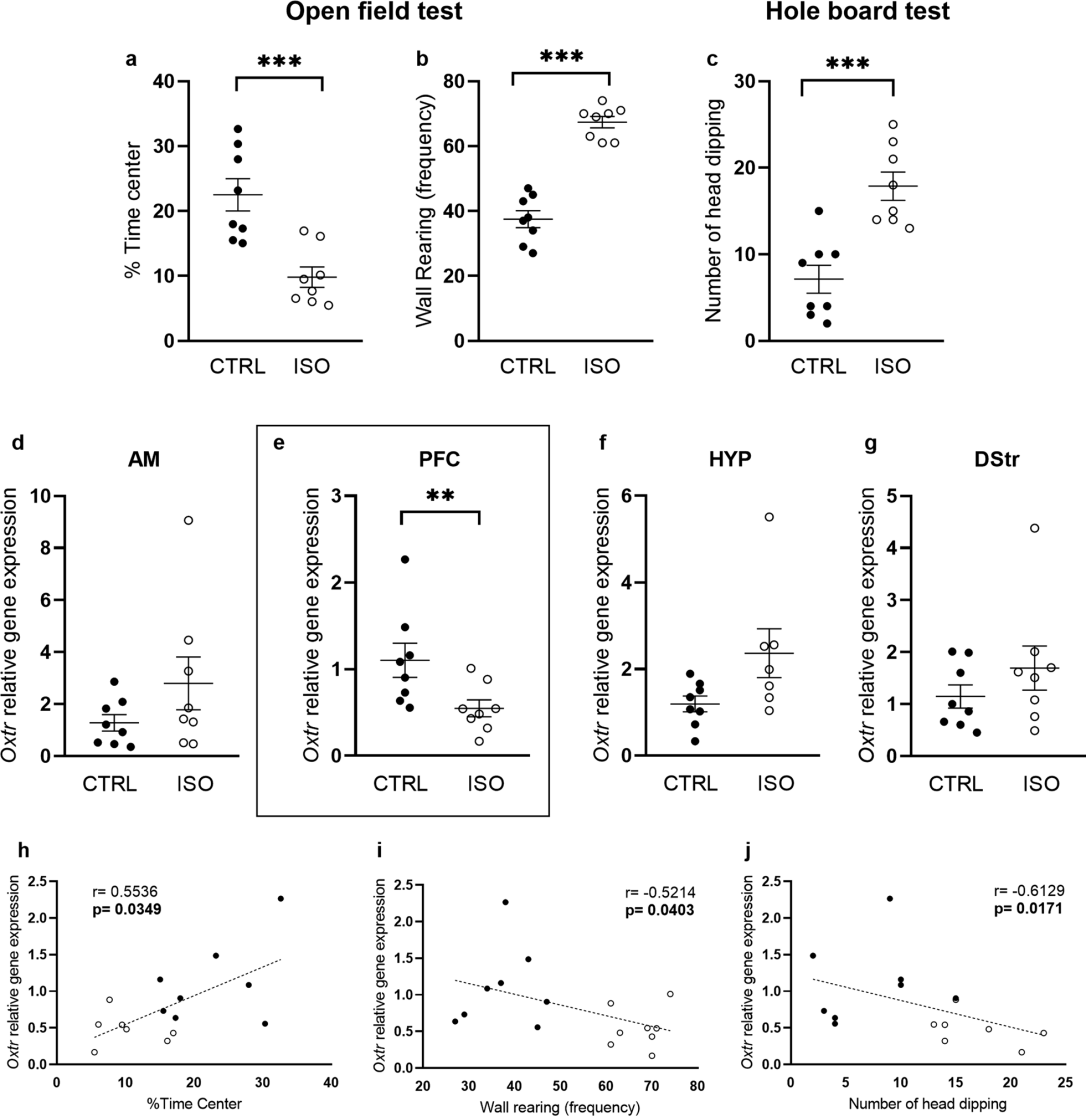
Behavioral and molecular analysis in ISO rats. **a** ISO rats (*n* = 8) housed individually for five weeks (from PND 21 to PND 54, ISO) showed an anxious-like phenotype in the open field test, as they spent less time in the center of the arena compared to CTRL animals (n = 8). Moreover, ISO rats showed stereotypic behaviors as they displayed a higher frequency of wall rearing (**b**) and more head dippings in the hole-board test (**c**); **d**–**g** *Oxtr* relative gene expression in the AM, PFC, HYP and DStr of ISO and CTRL rats. Scattered plots represent 2 ^(−DDCt)^ values calculated by the DDCt method [** *p* < 0.01]; **h**–**j** correlations between *Oxtr* expression and behavioral outcomes. Data were compared by Spearman's rank correlation coefficient

The two experimental groups did not differ in locomotor activity (crossing: *t* = 1.58, *p* = 0.13, *df* = 14; data not shown). The ISO rats showed an anxious-like phenotype compared to CTRL animals, as they spent less time in the center (*t* = 4.31, *p* < 0.001, *df* = 14; Fig. 5a) and more time on the peripheral parts of the arena compared to CTRL rats (*t* = 4.81, *p* < 0.001, *df* = 14; data not shown). Moreover, ISO rats showed stereotypic behaviors as they displayed a higher frequency of wall rearing (*t* = 9.56, *p* < 0.001, *df* = 14; Fig. 5b) compared to CTRL rats. There were no significant differences between ISO and CTRL rats in rearing frequency (*t* = 1.87, *p* = 0.081, *df* = 14; data not shown).

#### Hole-board test

The ISO rats showed stereotyped behaviors in the hole-board test; in fact, they made more head dippings (*t* = 4.69, *p* < 0.001, *df* = 14; Fig. 5c) compared to the CTRL group.

No differences were found between ISO and CTRL rats in the forced swim and elevated plus-maze tests (see Additional file 1: Fig. S5a–f).

#### Molecular analysis performed in brain tissues

Overall changes in *Oxtr* mRNA levels are reported in Fig. 5d–g. Statistical analysis showed that 5 weeks of social isolation determined selective changes of *Oxtr* gene expression in the prefrontal cortex (ISO: 0.55 ± 0.10, CTRL: 1.10 ± 0.20; *p* = 0.007, Fig. 5e). No significant differences between ISO and CTRL rats were observed in the other brain regions analyzed (Fig. 5d, f, g). Moreover, in the PFC, we report significant correlations between *Oxtr* gene expression changes and performance in the open field (time center percentage: Spearman's *r* = 0.554, *p* = 0.035; wall rearing frequency: Spearman's *r* = − 0.521, *p* = 0.040) and hole-board test (number of head dipping: Spearman's *r* = − 0.613, *p* = 0.017) (Fig. 5 h-j). No differences in DNA methylation of the *Oxtr* CpG island in the PFC of ISO rats with respect to controls (Additional file 1: Fig. S5g) as well as no correlation between the gene expression and DNA methylation were observed (Additional file 1: Fig. S5h).

#### Microbiota characterization in feces

**Fig. 6:**
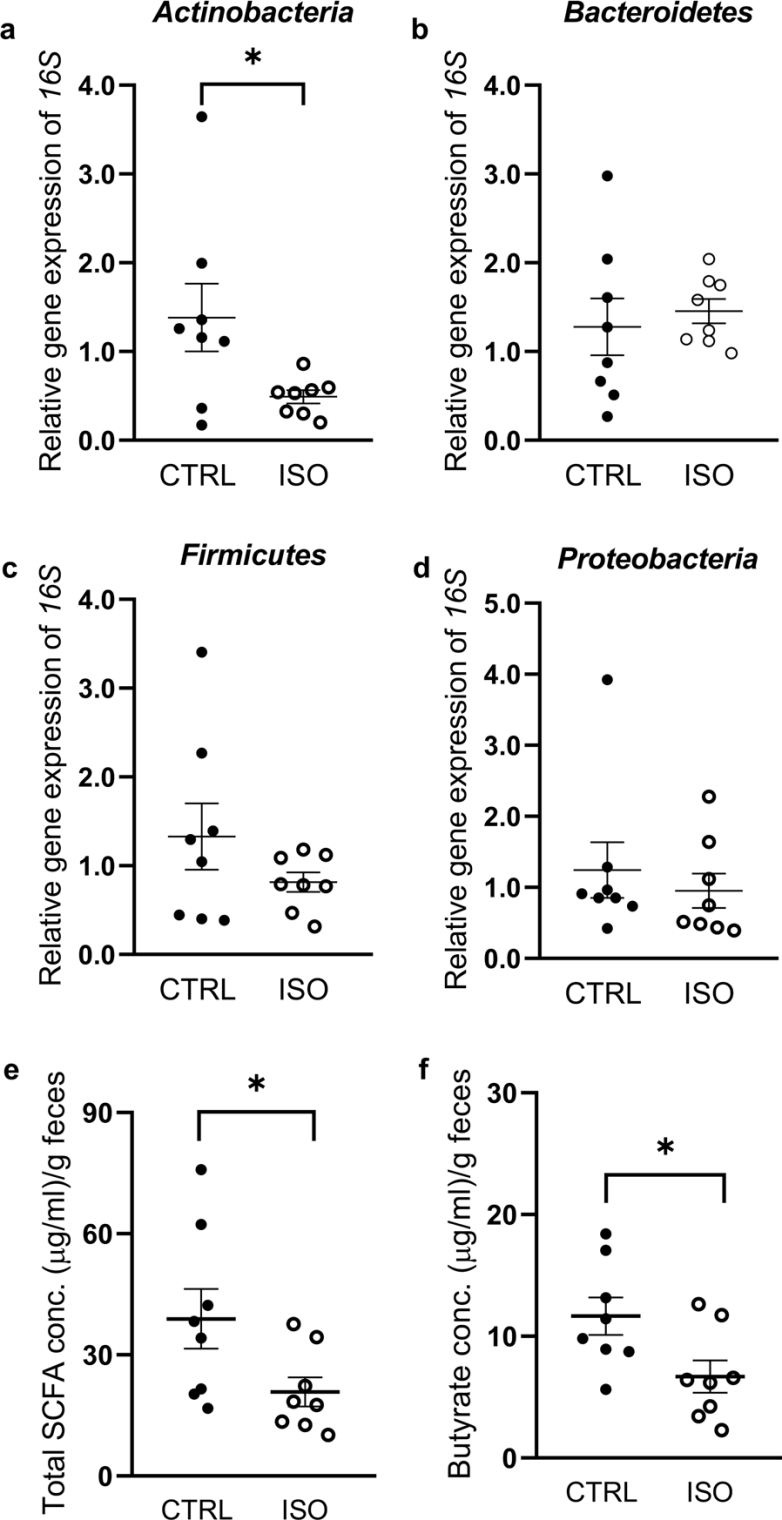
Relative abundance of the bacterial phyla (**a** Actinobacteria, **b** Bacteroidetes, **c** Firmicutes, **d** Proteobacteria) quantified in the feces of ISO (n = 8) and CTRL (n = 8) rats collected at social isolation time point T1. Scattered plots represent 2 ^(−DDCt)^ values calculated by the DDCt method. Concentration of fecal total SCFA (**e**) and butyrate (**f**) quantified using fecal samples collected at social isolation time point T1. ***p < 0.001, **p < 0.01, *p < 0.05 vs CTRL group

The levels of the main bacterial phyla are reported in Fig. 6a–d. A selective decrease was observed in *Actinobacteria* in ISO animals when compared to CTRL group (CTRL: 1.38 ± 0.38; ISO: 0.49 ± 0.07; *p* = 0.049) (Fig. 6a). No differences were observed in *Bacteroidetes* (CTRL: 1.28 ± 0.32; OCD: 1.45 ± 0.14; *p* = 0.46), *Firmicutes* (CTRL: 1.33 ± 0.37; OCD: 0.81 ± 0.11; *p* = 0.44) and *Proteobacteria* (CTRL: 1.24 ± 0.39; OCD: 0.95 ± 0.24; *p* = 0.48) (Fig. 6b–d), whereas it was not possible a quantitative detection of *Fusobacteria* in the rat stool by qPCR.

#### Evaluation of short chain fatty acid levels in feces

The SCFA content was quantified using fecal samples collected at different social isolation time points (T0, T1, T2 and T3) and measuring total SCFAs, as well as acetate, propionate, butyrate and valerate levels (Additional file 1: Table S7). Total SCFA levels were significantly reduced one week after isolation (T1) in ISO rats feces when compared to CTRL group (CTRL: 38.92 ± 7.42; ISO: 20.83 ± 3.59; *p* = 0.049) (Fig. 6e). Moreover, a significant decrease of butyrate concentration was observed in the same experimental group at the same time point (CTRL: 11.65 ± 1.54; ISO: 6.69 ± 1.35; *p* = 0.049) (Fig. 6f), whereas acetate, propionate and valerate levels were not affected by social isolation. Consistently, a correlation between SCFA levels and the behavioral outcomes was also observed considering both experimental groups at T1 (Additional file 1: Table S8). Further data analysis is reported in Additional file 1: Section (Additional file 1: Tables S9 and S10).

## Discussion

The first main outcome of this investigation is that we observe an altered transcriptional regulation of the *OXTR* gene in biological samples from OCD patients when compared to healthy controls. OXTR binds to its endogenous nonapeptide ligand oxytocin that is associated with several social behaviors and emotional regulation as well as to mood disorders [53–55]. The gene coding for OXTR, located on human chromosome 3p25.3 [56, 57], is expressed both in the brain and within peripheral organs. The *OXTR* gene spans 17 kilobytes (kb) and contains 3 introns and 4 exons. Exons 1 and 2 correspond to non-coding regions, while exons 3 and 4 encode for the OXTR involved in the activation of different second messengers in the cell [58]. Moreover, we report higher DNA methylation levels at specific CpG sites in exon 3 of *OXTR* in PBMCs collected from OCD patients, when compared to controls. A similar hypermethylation in blood collected from OCD patients has been already reported a few years ago [17], and more recently by Schiele et al. [18].

Several environmental factors, such as stress and adverse early life experiences, have been blamed for causing OCD [59–62], and remarkably *OXTR* DNA methylation at exon 3 has been suggested to be responsive to the environment [63–65]. For instance, higher DNA methylation at two CpG sites located within exon 3 has been reported in child abused [63]. Trier social stress test induces rapid increase in *OXTR* DNA methylation, again within exon 3, going back to normal levels 90 min after the test [64]. Adverse early life experience, as low childhood maternal care, has also been associated with increased *OXTR* exon 3 DNA methylation in adults [65]. Moreover, besides OCD, *OXTR* DNA methylation at exon 3 has been associated with other central nervous system disorders such as anxiety disorder [66], autism spectrum disorder [67], depression [68] and psychopathy [69]. Among the CpG sites we here investigated, we report, in particular, a key role for the one we named CpG site 4. Of interest, Unternaehrer and colleagues reported for the same CpG site an increase in DNA methylation evoked by stress [64]. Of note, based on PROMO database (http://alggen.lsi.upc.es/cgi-bin/promo_v3/promo/promoinit.cgi?dirDB=TF_8.3), it is possible to identify putative transcription factors binding to this CpG site, such as T3R-beta1, TFII-I, STAT4, E2F-1, c-Ets-1, Elk-1 and GR-alpha. Further studies are needed to specifically define their role in *OXTR* gene regulation. However, it is also needed to address whether an altered DNA methylation status might become fixed or respond to new and positive environmental stimuli over time, and this could be of particular relevance for *OXTR* gene level in OCD when alterations are evoked during developmentally sensitive time-periods.

Others already reported reduction in *OXTR* gene expression in patients with mental disorders, for instance a decrease in autism cases [67] and in schizophrenia patients [70] in brain regions involved in social cognition. *OXTR* expression was found significantly higher also in peripheral blood lymphocytes of first episode schizophrenia patients [71], and in the dorsolateral prefrontal cortex of individuals with major depression and bipolar disorder [72].

The same pattern of changes in the epigenetic mark occurs in DNA obtained from saliva samples of OCD patients and healthy controls, and there is a significant correlation between these alterations and those observed in blood. Altered DNA methylation in saliva of OCD patients, yet at different CpG sites at *OXTR* gene (specifically in intron 1), was also observed very recently by others [20]. We thus confirm previous reported *OXTR* hypermethylation at exon 3 in OCD patients in blood and observed for the first time this epigenetic modulation in saliva. It should be mentioned that the correlation between blood and saliva DNA methylation has not always been reported, due to the high variability in individual cellular composition, even at different time points [73], overall affecting the proportion of white blood cells and buccal keratinocytes [74, 75]. However, the use of saliva samples to evaluate DNA methylation as biomarker has been already suggested, for example, for borderline personality disorder [76], schizophrenia [77], stress [78] or as a predictor of childhood obesity [79]. In terms of epigenetic modifications, it has been reported that there is higher correlation between saliva and brain tissues when compared to brain and blood [80].

Moreover, we show that alterations in the epigenetic mark seem to be larger in women then in men, an observation that is in line with other studies reporting a higher sensitivity in female compared to male subjects along with a sex-specificity of the OXT system [81]. There is also a positive correlation between the increase in the levels of the epigenetic mark and the age in OCD patients, but not in healthy controls, as well as the years from OCD onset in patients but not the rate of severity measured by Y-BOCS. These data might suggest that alteration of the molecular outcome is connected with the progression of the disease but not with its severity. Finally, in subjects under any pharmacological treatment the epigenetic mark was not affected when compared to drug-naïve patients, in agreement with a previous report by Cappi and co-workers [17].

Last but not least, we analyzed the microbiota in OCD saliva samples, specifically focusing on the expression of the major phyla. Nowadays dysregulation of the gut–brain axis has been recognized as a new area of research to better understand the pathophysiology of mental illnesses [82–90]. In the same way, the human oral microbiota has become a new research area aimed at promoting the progress of disease diagnosis, complementing disease treatment, and developing personalized medicines. Disturbance to the oral microbiota has been found to be associated not only with infectious oral diseases but also with systemic diseases [91], and it might function as potential biomarker for several human conditions [41, 91–95]. Bacteria in the mouth can reach the brain under particular conditions, for instance when permeability of the blood–brain barrier is higher [96], thus exposing the brain to bacterial metabolites [97]. We here show a selectively higher relative abundance in *Actinobacteria* phylotype in saliva samples from OCD patients, when compared to healthy controls, without changes for the other phyla analyzed. Similar alterations in saliva *Actinobacteria* levels have been previously reported in children with autism spectrum disorder [98], and increased levels of *Actinobacteria* were also reported in fecal material of patients suffering from major depressive disorder [99–101] and bipolar disorder [101] patients, when compared to controls.

We also show a lower *Fusobacteria* to *Actinobacteria* ratio in OCD cases when compared to controls, consistently with what it has been very recently reported [102]. Within the phylum of *Actinobacteria*, a relevant role is played by *Bifidobacteria* known to produce folate which, beside its relevance in many metabolic pathways, is crucial for the production of *S*-adenosylmethionine. The latter in turn is a methyl-donating substrate for DNA methyltransferases [103], and thus, it contributes to DNA methylation [104]. Interestingly, we here report a significant correlation between the increase in DNA methylation at *OXTR* gene and that in the expression of *Actinobacteria*. We also observed higher levels of *Firmicutes* phylotype in OCD subjects compared to controls, albeit to a lesser extent. However, no differences between the two groups were observed in *Firmicutes/Bacteroidetes* ratio, widely accepted as relevant in maintaining normal intestinal as well as mouth homeostasis [105]. Moreover, these changes were not correlated with the alterations observed in OXTR DNA methylation, differently from what observed for *Actinobacteria*. These preliminary results add information to the relationship between host epigenome and gut microbiome, suggesting how they might influence each other and might impact on OCD.

In line with the clinical data, we also evaluated the role of central *Oxtr* transcriptional regulation in rats socially isolated for 5 weeks that showed stereotyped behaviors reminiscent of OCD. Alterations in the oxytocinergic system might thus be induced by social isolation, and this also resulted in anxious and somewhat stereotypic, OCD-like behaviors. Besides peripheral release, oxytocin acts within the brain as a neurotransmitter playing a pivotal role in numerous social behaviors [106–110]. Consistently with the human data, we report the down-regulation of *Oxtr* gene expression selectively in the PFC of socially-isolated rats and this alteration correlates with anxiety-like as well as stereotyped behaviors displayed by the animals. The PFC abundantly expresses *Oxtr* [111–114] and contains oxytocin sensitive neurons [115], receiving projections from the hypothalamus [116, 117]. Moreover, a brain imaging study reported higher *Oxtr* methylation associated with increased brain activity in cortical areas involved in social perception [118]. Interestingly, we also observe a reduction in feces levels of both butyrate and total SCFA at early stages of the social isolation procedure and these are correlated with the alterations we observe in *Oxtr* gene expression, even if not significantly. Butyrate inhibits histone deacetylases (HDACs) activity; thus, it might be hypothesized that, in socially isolated rats where butyrate is reduced, the involvement of another epigenetic mark, with the recruitment of HDACs, might be involved contributing to the reduction in the transcriptional activity. We also report a reduction in rat feces of actinobacteria, known to promote butyrate production [119]. It should be noticed that this microbiota modulation in rat feces is opposite to the one observed in human saliva. This might be due of course to the different matrix as well to the different organism investigated and it would reflect the potentially different epigenetic mechanism involved as we here propose. In fact, in rats we do not observe any change in gene DNA methylation and this might support what stated above.

## Study limitations

One limitation of the present study is that post-weaning social isolation is not an animal model of OCD [120]. Studies showed that social isolation in rodents has a negative impact on cognitive flexibility [121–124]), even if others reported increased cognitive flexibility in rats socially isolated [125]. However, since our aim was to address epigenetic regulation of gene transcription, we decided to use socially isolated rats as an environmental animal model, where we definitively observed certain behavioral abnormalities, such as stereotypic-like behaviors in the hole-board test, which anyhow may only be evocative of certain behavioral symptoms showed by OCD patients. Another limitation of our study is that we did not use female rats. However, most of the studies on the impact of social isolation on oxytocin system have been carried out on male rodents [126], further investigations would be needed to address potential sex-related differences.

Regarding human data, future studies are needed to investigate the possible impact on the microbiome of confounding factors. Indeed, the microbiome has shown to be influenced by dietary habits, smoking, body mass index, physical exercise and lifestyle factors [127] that have not been assessed in the present study. Moreover, it would be interesting to compare patients with different OCD phenotypes (not recorded in the present study), considering their potential indirect impact on the microbiome (e.g., patient with contamination obsessions might have particular food restrictions that ultimately influence the microbiome).

## Conclusions

Our data confirm the relevance of *OXTR* gene regulation in OCD and support the possible role of microbiota-derived metabolites as potential biomarkers for early diagnosis and prognosis of OCD as already suggested for other diseases [128]. The use of saliva samples to collect genomic material in OCD patients is a relevant strategy allowing an easier analysis also considering different ages (children, elderly) and/or sensitive subjects. It is indeed important to recall that OCD is characterized by contamination fears and that associated with blood withdrawal is often present [129]. Moreover, the use of saliva would allow to easily collect multiple samples at different time points [130, 131], giving the opportunity to monitor the development of the disease. Finally, the animal data definitely corroborate the clinical ones at central level and further point out to a possible microbiota-host epigenetic axis.

## Supplementary Information

**Additional file 1.** Supplementary material: methods, results, tables and figures.

## Data Availability

The data used and/or analyzed during the current study are available from the corresponding author on request.

## Abbreviations

OCD: Obsessive–compulsive disorder
OXTR: Oxytocin receptor gene
GABR1: Gamma-aminobutyric acid type B receptor subunit 1
MOG: Myelin oligodendrocyte glycoprotein
BDNF: Brain-derived neurotrophic factor
SLC6A4: Serotonin transporter
SCID: Semi-structured interview based on DSM-5 criteria
PND: Postnatal day
ISO: Social isolation
T0: One day before isolation
T1: One week after isolation
T2: Three weeks after isolation
T3: Five weeks after isolation
PBMC: Peripheral blood mononuclear cell
PFC: Prefrontal cortex
AM: Amygdala
HYP: Hypothalamus
DStr: Dorsal striatum
SCFA: Short chain fatty acid
CpG: Cytosine-phosphate-guanine
