# Supplementary material for: Regulation of oxytocin receptor gene expression in obsessive–compulsive disorder: a possible role for the microbiota-host epigenetic axis
Source: Clin Epigenetics. 2022 Mar 31;14:47. doi: 10.1186/s13148-022-01264-0 (PMC8973787; doi:10.1186/s13148-022-01264-0)
Supplement: Supplementary file 1 — Additional file 1. Supplementary material: methods, results, tables and figures. [file 13148_2022_1264_MOESM1_ESM.docx]

*Supplementary material*

***Methods***

***Animal Behavioral Tests***

- ***Open Field Test***

The open field apparatus consisted of a square arena (40 (length) x 40 (width) x 60 (height) cm). Each animal was individually placed in the central zone of the apparatus and allowed to explore for 10 min. After each session, the apparatus was cleaned with ethanol 70% [1]. Each session was recorded with a camera positioned above the apparatus for subsequent behavioral analysis performed using the Observer 3.0 software (Noldus Information Technology). The following parameters were analyzed: the number of crossings (a grid, dividing the arena into equally sized squares (6 x 6 cm), was projected over the recordings, and the number of line crossings made by the animal was recorded), the frequency of rearing (defined as the rat standing on the hindlimbs without touching the wall) and wall rearing behavior (defined as the standing on the hindlimbs and touching the wall) and the time spent in the central (15 cm × 15 cm square) and side (less than 5 cm away from the walls) areas of the arena. The percentage of time spent in the center arena was calculated as (time center/600 sec) x 100.

- ***Hole-board test***

The hole-board test is widely used test to assess stereotyped and repetitive behaviors [1]. The apparatus was a gray square metal table (40 (length) × 40 (width) × 10 (height) cm) with 16 evenly spaced holes (4 cm in diameter), inserted in a Plexiglas arena (40 (length) × 40 (width) × 60 (height) cm). The test was performed as previously described [2]. The rats were individually placed in the apparatus and their behavior was observed for 5 min. Head dipping behavior was scored by the number of times an animal inserted its head into a hole at least up to the eye level. Each session was recorded with a camera positioned above the apparatus for subsequent behavioral analysis performed using the Observer 3.0 software (Noldus Information Technology, The Netherlands).

- ***Elevated plus-maze in rats***

The elevated plus-maze test is one of the most commonly used tests to detect anxiety-like behaviors. The apparatus comprised two open and two closed arms (50 (length) × 10 (width) × 40 (height) cm) that extended from a common central platform (10 (length) × 10 (width) cm). The rats were individually placed on the central platform of the maze for 5 min. Each session was recorded with a camera positioned above the apparatus for subsequent behavioral analysis performed using the Observer 3.0 software (Noldus Information Technology). The following parameters were analyzed [1]:

1. % time spent in the open arms (% TO): (seconds spent on the open arms of the maze/seconds spent on the open + closed arms) × 100;

2. % open-arm entries (% OE): (the number of entries into the open arms of the maze/number of entries into open + closed arms) × 100;

3. Frequency of stretched attend posture (SAP).

- ***Forced swim test in rats***

The forced swim test (FST) is one of the most commonly used behavioral tests for assessing depressive-like behaviors. In two sessions separated by 24 hr, rats were forced to swim in a narrow cylinder from which they could not escape, filled with tap water at 23 ± 1 °C. The first day (pre-test) each rat was placed in the water filled cylinder container for 15 min. After 15 minutes had elapsed, the rat was removed from the container and placed in a cage for transient drying with the heating lamp above and the heating pad below it. The water was changed after each session to avoid any influence on the next experimental animals. Twenty-four h later, the rat was placed in the cylinder container filled with water for a 5-min test session. After the test session, all the procedures performed after the training were replicated.

Each session was recorded with a camera positioned above the apparatus for subsequent behavioral analysis performed using the Observer 3.0 software (Noldus Information Technology). The following parameters were analyzed: 1) the time spent by the animal in “climbing”, intended as quick movements of the forelimbs, 2) the time spent by the animal in “floating”, intended as the absence of any movement except for those necessary for keeping the nose above water and 3) the time spent by the animal in “swimming”, intended as movement of forelimbs or hind limbs in a paddling fashion [3].

***Analysis of gene expression***

RevertAid H Minus First Strand cDNA Synthesis kit (Thermo Scientific, Waltham, MA, USA) was used to convert one µg of total RNA into cDNA. Real-time quantitative polymerase chain reaction (RT-qPCR) was performed using SensiMix SIBR Low-ROX Kit (Bioline Reagents, London, UK) on a DNA Engine Opticon 2 Continuous Fluorescence Detection System (MJ Research). The relative amount of *OXTR* mRNA in human and rat samples was first normalized to four endogenous reference genes glyceraldehyde-3-phosphate dehydrogenase (GAPDH), beta-actin (BACT), cytochrome C oxidase subunit 6A1 (COX6A1) and ribosomal protein lateral stalk subunit P0 (RPLP0), then was calculated by the Delta-Delta Ct (DDCt) method and converted to relative expression ratio 2^(-DDCt)^ for statistical analysis [4,5]. See primers sequences in supplementary table S1.

***Analysis of DNA methylation***

As previously described [6] 500 ng of DNA from each purified sample was subjected to bisulfite modification using the EZ DNA Methylation-GoldTM Kit (Zymo Research, Orange, CA, USA), inducing chemical conversion of unmethylated cytosine residues to uracil. The DNA methylation status of each CpG site in *OXTR* CpG island was assessed using a pyrosequencing assay.

DNA, after bisulfite treatment, was first amplified by PyroMark PCR Kit (Qiagen, Hilden, Germany) with a biotin labeled primer (Hs_OXTR_01_PM PyroMark CpG assay, PM00016821; Rn_Oxtr_02_PM PyroMark CpG assay, PM00546546) according to the manufacturer’s recommendations. PCR conditions were as follows: 95°C for 15 min, followed by 45 cycles of 94°C for 30 s, 57°C for 30 s, 72°C for 30 s, and, finally, 72°C for 10 min. Specificity of PCR products was then verified by electrophoresis. The sequencing was performed on a PyroMark Q24 ID using Pyro Mark Gold reagents (Qiagen, Hilden, Germany), after immobilizing the amplified product to Streptavidin Sepharose High-Performance (GE Healthcare, Chicago, IL, USA) beads via biotin affinity and denatured to allow the annealing with the sequencing primer. DNA methylation level was analyzed through the PyroMark Q24 ID version 1.0.9 software which calculates the methylation percentage mC / (mC + C) (mC = methylated cytosine, C = unmethylated cytosine) for each CpG site, allowing quantitative comparisons. Quantitative methylation results were expressed both as a percentage of every single CpG site and as the average of the methylation percentage of all the 4 CpG sites under study.

***Microbiota analysis***

Bacterial DNA from rat feces was isolated using TRIzol reagent (Thermo Fisher Scientific) according to the specifications of the instructions with some modifications in order to avoid protein contaminations. qPCR was used to quantify major phyla abundance using primers targeting the 16S rRNA gene phyla-specific normalized to the universal bacterial 16S rRNA sequence (detailed sequences reported in [7,8]. An amount of 20 ng of gDNA extracted from human saliva and rat feces was used for the reaction. PCR conditions were as follows: 5 min at 95°C, 50 cycles of 95°C for 20 s, 57°C for 10 s and 72°C for 15 s, and a final incubation of 5 min at 72°C. Differences in threshold cycle number were used to quantify the relative amount of the PCR targets contained within each tube. Quantification of phyla-specific gene abundance was performed using the DDCt method and converted to relative expression ratio 2^(-DDCt)^ for statistical analysis [4]. See primers sequences in supplementary table S1.

***Evaluation of Short Chain Fatty Acid levels***

All reagents were of purest analytical grade purchased from Sigma-Aldrich; Waters, Acetonitrile, Isopropanol and Formic acid, all LC-MS grade are purchased from Sigma-Aldrich. Supernatants collected after centrifugation were diluted 20-fold in H_2_O/IsoP 30/70 (v/v) solution and 50 μL of diluted sample were mixed with 50 μL of internal standard (IS) solution (acetic acid-d4, 100 μg/mL); then 20 μL of 3-nitrophenylhydrazine HCl (NPH) 200mM and 20 μL of 120 mM N-(3-dimethylaminopropyl)-N0-ethylcarbodiimide HCl (EDC HCl)– 6% pyridine solution were added and mixed for 30 min at 40 °C; the reaction was stopped with 200 μL of 0.1% HCOOH. After derivatization, samples were centrifuged at 10,000 g for 10 min at 4 °C and consequently they were analyzed by HPLC-MS/MS analysis.

Analyses were performed by API2000 triple quadrupole mass spectrometer from Sciex (Toronto, ON, Canada) coupled with PE Series 200 LC micro pump system (Perkin Elmer, Norwalk, CT, USA) equipped with ACE Excel C18-PFP column (100x2.1 mm) packed with 2.0 μm particles (Advanced Chromatography Technologies, Aberdeen, UK); a guard column was used for column safety. The mobile phases were selected in order to obtain the complete separation of the analytes: phase A was H2O 0.1% HCOOH and phase B was ACN 0.1% HCOOH; flow rate was set at 0.25 mL/min, with injected volume set at 5 μL. Elution of the analytes was achieved using the following gradient: chromatographic run started at 10% of B with linear increase to 100% B in 10 min, then it was kept for 2 min at 100% of B, at the end phase B was decreased to 10% in 1 min and column was equilibrated in 2 min.

The MS/MS acquisition was performed in Multi Reaction Monitoring (MRM) mode, by selecting 2 molecular ion/fragment ion transitions for each analyte; ionization was performed by Electrospray (ESI) in negative mode, using a capillary voltage (IS) of −4200 eV, curtain gas (CUR) set at 20 psi, collision gas (CAD) at 6 psi, source temperature set at 350 °C (TEM), with Ion Source Gas1 (GS1) and Gas2 (GS2) both set at 30 and 40 psi respectively. All source and instrument parameters for the monitored analytes were tuned by injecting standard solutions at a concentration of 100 ng/mL at 7 μL/min by a syringe pump.

Peaks areas were detected and quantified through MultiQuant 3.0 Software (Version 3.0.5373.0, 2013 Sciex).

***Method Validation for the Evaluation of SCFA levels***

A validation protocol of the method was performed: accuracy, precision, limits of quantification (LOQs) and linearity were evaluated with the following standard SCFAs: Acetic Acid, Propionic Acid, Butyric Acid, Valeric Acid. A mixed standard solution containing each of the 4 SCFAs was prepared in 50:50 (v/v) acetonitrile:water and derivatization followed as described for fecal samples (see materials and methods). Calibration curves with 7 different standard SCFA concentrations ranging from 0.5 to 50 μg/mL were set for analysis. (a sample run is reported in figure S2). All MRM parameters are shown in Table S2.

Accuracy was tested by spiking the analytes and calculated by blank subtraction method and expressed as bias; precision was calculated on intraday replicates of the same sample and expressed as relative standard deviation (RSD%); LOQs were determined on calibration curves as the concentration with at least a signal to noise ratio of 10. Calibration curves were derived by plotting the peak area of analytes to IS versus the concentration using a least squares regression model. The squared correlation coefficient (R^2^) was used to estimate linearity. Results are shown in Table S3.

***Results***

***Forced swim test***

No differences were found between ISO and CTRL rats in the forced swim test. Indeed, a Student’s t-tests performed on the parameters measured in this test gave the following results: climbing time (t=0.75, p=0.46, df=14; Supplementary Figure S4A); floating time (t=0.62, p=0.54, df=14; Supplementary figure S4B); swimming time (t= 1.23; p=0.23; df=14; Supplementary figure S4C).

***Elevated plus-maze test***

No differences were found between ISO and CTRL rats in the elevated plus-maze test. Indeed, a Student’s t-tests performed on the parameters measured in this test gave the following results: percentage of time spent in the open arms (t=0.88, p=0.39, df=13; Supplementary Figure S4D); percentage of open-arm entries t=0.40, p=0.69, df=13; Supplementary figure S4E); frequency of SAP (t= 1.1; p=0.29; df=13; Supplementary figure S4F).

***Tables***

**Table S1:** List of primers used for gene expression analysis.

|  | Gene | Sequence | |
| --- | --- | --- | --- |
|  |  | **Forward** | **Reverse** |
| *Human* | *OXTR* | CGCCCAAGGAAGCCTCG | CACCTCTTCCACGAACTCGT |
|  | *GAPDH* | CAGCCTCAAGATCATCAGCA | TGTGGTCATGAGTCCTTCCA |
|  | *β -ACT* | GACCCAGATCATCAGCA | CCATCACGATGCCAGTGG |
|  | *COX6A1* | CATCAGGACCAAGCCGTTTCC | ATGTGCAGAGTAACGGTCCA |
|  | *RPLP0* | TCCTCGTGGAAGGCCCG | TGCCACGCAGGGTTTAAAGA |
|  |  |  |  |
| *Rat* | *Oxtr* | CAAGGAAGCTTCTGCCTTCAT | CTGCACGAGTTCGTGGAAGA |
|  | *Gapdh* | AGACAGCCGCATCTTCTTGT | CTTGCCGTGGGTAGAGTCAT |
|  | *β-Act* | AGATCAAGATCATTGCTCCTCCT | ACGCAGCTCAGTAACAGTCC |
|  |  |  |  |
| *Bacteria* | *Universal 16S rRNA* | AAACTCAAAKGAATTGACGG | CTCACRRCACGAGCTGAC |
|  | *Bacteroidetes* | AAACTCAAAKGAATTGACGG | GGTAAGGTTCCTCGCGCTAT |
|  | *Firmicutes* | TGAAACTYAAGGAATTGACG | ACCATGCACCACCTGTC |
|  | *Actinobacteria* | TACGGCCGCAAGGCTA | TCRTCCCCACCTTCCTCCG |
|  | *Proteobacteria* | TCGTCAGCTCGTGTYGTGA | CGTAAGGGCCATGATG |
|  | *Fusobacteria* | GGATTTATTGGGCGTAAAGC | GGCATTCCTACAAATATCTACGAA |

**Table S2**. Location of the CpG sites under study at human and rat *OXTR* genes. Genome assembly GRCh38.p13, transcript OXTR-201 (ENST00000316793.8) for human. Genome assembly Rnor_6.0, transcript Oxtr-201 (ENSRNOT00000007724.2) for rat.

| **Human *OXTR* CpG Island (chromosome 3)** | | | | | | | | | |
| --- | --- | --- | --- | --- | --- | --- | --- | --- | --- |
| **CpG Site 1** | | **CpG Site 2** | | | **CpG Site 3** | | | **CpG Site 4** | |
| 3: 8767851 | | 3: 8767864 | | | 3: 8767870 | | | 3: 8767878 | |
| **Rat *Oxtr* CpG Island (chromosome 4)** | | | | | | | | | |
| **CpG Site 1** | **CpG Site 2** | | **CpG Site 3** | **CpG Site 4** | | **CpG Site 5** | **CpG Site 6** | | **CpG Site 7** |
| 4: 144416131 | 4: 144416105 | | 4: 144416103 | 4: 144416099 | | 4: 144416097 | 4: 144416093 | | 4: 144416091 |

**Table S3.** MRM instrumental parameters of the selected analytes

| ID | Q1 (m/z) | Q3 (m/z) | DP (eV) | EP (eV) | CE (eV) | CXP (eV) |
| --- | --- | --- | --- | --- | --- | --- |
| Acetic acid | 194.1 | 137.1 | -46 | -7 | -20 | -11 |
|  |  | 151.5 |  |  | -30 | -11 |
| Propionic acid | 208.0 | 137.1 | -53 | -10 | -16 | -5 |
|  |  | 165.1 |  |  | -30 | -9 |
| Butyric acid | 222.0 | 137.1 | -50 | -10 | -20 | -5 |
|  |  | 179.1 |  |  | -30 | -4 |
| Valeric acid | 236.1 | 137.1 | -38 | -10 | -24 | -5 |
|  |  | 157.1 |  |  | -30 | -10 |
| Acetic acid-d4 | 198.1 | 138.1 | -35 | -7 | -28 | -6 |

**Table S4.** Validation results

| ID | IS | RT(min) | LOQs(μg/mL) | Acc(%) | RSD(%) | Eq | R^2^ |
| --- | --- | --- | --- | --- | --- | --- | --- |
| Acetic acid | Acetic acid-d4 | 4.92 | 0.1 | ±6% | 5% | Y=0.24802X-0.0009 | 0.9977 |
| Propionic acid | Acetic acid-d4 | 5.20 | 0.06 | ±4% | 8% | Y=0.76159X+0.40492 | 0.9975 |
| Butyric acid | Acetic acid-d4 | 5.45 | 0.04 | ±5% | 11% | Y=1.74071X+0.56464 | 0.9962 |
| Valeric acid | Acetic acid-d4 | 5.76 | 0.01 | ±9% | 12% | Y=2.14883X+2.67553 | 0.9951 |

**Table S5.** Spearman correlation analysis between the percentage of DNA methylation at *OXTR* exon III (AVE) in both PBMCs and saliva, and the age of the subjects under study considering the overall population, as well as OCD subjects and healthy controls (CTRL) separately. Significant *p value < 0.05.

| **% DNA Methylation *OXTR* exon III (AVE)** | **Age (Y.O.)** | |
| --- | --- | --- |
| **PBMCs** | **Spearman’s r** | **p value** |
| Overall | 0.166 | 0.250 |
| OCD | 0.272 | 0.153 |
| CTRL | 0.057 | 0.810 |
| **Saliva** | **Spearman’s r** | **p value** |
| overall | 0.285 | **0.015*** |
| OCD | 0.338 | **0.029*** |
| CTRL | 0.108 | 0.564 |

**Table S6.** Spearman correlation analysis between the percentage of DNA methylation at *OXTR* exon III (AVE) and Y-BOCS scores in OCD subjects also stratified by gender. Significant *p value < 0.05.

| **% DNA Methylation *OXTR* exon III (AVE)** | **Y-BOCS value** | |
| --- | --- | --- |
| **PBMCs** | **Spearman’s r** | **p value** |
| OCD | -0.236 | 0.209 |
| Women | -0.009 | 0.979 |
| Men | -0.493 | 0.054 |
| **Saliva** | **Spearman’s r** | **p value** |
| OCD | 0.163 | 0.314 |
| Women | 0.356 | 0.124 |
| Men | 0.095 | 0.690 |

**Table S7.** SCFA concentrations expressed as (µg/ml)/g feces obtained from each animal at different social isolation time points. Mean and Standard Error of Mean for control and ISO individuals are reported. Significant differences in SCFA concentrations were highlighted (*****p<0.05).

|  | **TOTAL SCFA (µg/ml)/g feces** | | | |
| --- | --- | --- | --- | --- |
|  | T0 | T1 | T2 | T3 |
| ***CTRL*** | 21.03 ± 2.89 | 38.92 ± 7.42 | 44.49 ± 3.37 | 40.58 ± 2.03 |
| ***ISO*** | 20.86 ± 6.77 | **20.83 ± 3.59** ***** | 30.97 ± 4.30 | 34.99 ± 4.92 |
|  | **ACETATE (µg/ml)/g feces** | | | |
|  | T0 | T1 | T2 | T3 |
| ***CTRL*** | 4.04 ± 0.55 | 9.63 ± 2.25 | 12.76 ± 0.92 | 9.24 ± 0.55 |
| ***ISO*** | 2.86 ± 0.51 | 5.62 ± 0.73 | 8.71 ± 1.07 | 7.52 ± 0.94 |
|  | **PROPIONATE (µg/ml)/g feces** | | | |
|  | T0 | T1 | T2 | T3 |
| ***CTRL*** | 9.90 ± 1.69 | 16.83 ± 4.59 | 16.63 ± 1.19 | 16.48 ± 1.07 |
| ***ISO*** | 8.63 ± 2.24 | 8.101 ± 1.75 | 11.53 ± 1.64 | 13.68 ± 1.99 |
|  | **BUTYRATE (µg/ml)/g feces** | | | |
|  | T0 | T1 | T2 | T3 |
| ***CTRL*** | 6.73 ± 0.73 | 11.65 ± 1.54 | 14.15 ± 2.51 | 13.64 ± 1.40 |
| ***ISO*** | 8.99 ± 4.55 | **6.69 ± 1.35 *** | 10.15 ± 1.88 | 12.70 ± 2.55 |
|  | **VALERATE (µg/ml)/g feces** | | | |
|  | T0 | T1 | T2 | T3 |
| ***CTRL*** | 0.36 ± 0.14 | 0.81 ± 0.25 | 0.94 ± 0.16 | 1.21 ± 0.09 |
| ***ISO*** | 0.37 ± 0.19 | 0.29 ± 0.12 | 0.59 ± 0.11 | 1.08 ± 0.20 |

**Table S8.** Spearman correlation analysis between SCFAs levels (total and butyrate) at T1 and behavioral outcomes. Significant *p value < 0.05.

|  | **Total SCFA conc. (μg/mL)/g feces** | |
| --- | --- | --- |
|  | **Spearman’s r** | **p value** |
| **% time center** | 0.491 | 0.055 |
| **Wall rearing** | -0.524 | **0.039*** |
| **Number head dipping** | -0.534 | **0.042*** |
|  | **Butyrate conc. (μg/mL)/g feces** | |
|  | **Spearman’s r** | **p value** |
| **% time center** | 0.517 | **0.042*** |
| **Wall rearing** | -0.524 | **0.039*** |
| **Number head dipping** | -0.546 | **0.031*** |

**Table S9.** Spearman correlation analysis between SCFAs levels (total and butyrate) at T1 and *Oxtr* relative gene expression. *p value < 0.05.

|  | ***Oxtr* relative gene expression** | |
| --- | --- | --- |
| **Total SCFA conc. (μg/mL)/g feces** | **Spearman’s r** | **p value** |
| overall | -0.111 | 0.695 |
| ISO | -0.357 | 0.444 |
| **Butyrate conc. (μg/mL)/g feces** | **Spearman’s r** | **p value** |
| overall | -0.186 | 0.507 |
| ISO | -0.678 | 0.109 |

**Table S10**. Spearman correlation analysis between relative abundance of Actinobacteria and *Oxtr* relative gene expression, and SCFAs levels (total and butyrate) at T1 expressed. Significant *p value < 0.05.

|  | **Relative abundance of Actinobacteria** | |
| --- | --- | --- |
|  | **Spearman’s r** | **p value** |
| ***Oxtr* relative gene expression** | 0.559 | **0.026*** |
| **Total SCFA conc. (μg/mL)/g feces** | 0.629 | **0.032*** |
| **Butyrate conc. (μg/mL)/g feces** | 0.825 | **0.001*** |

***Figures***


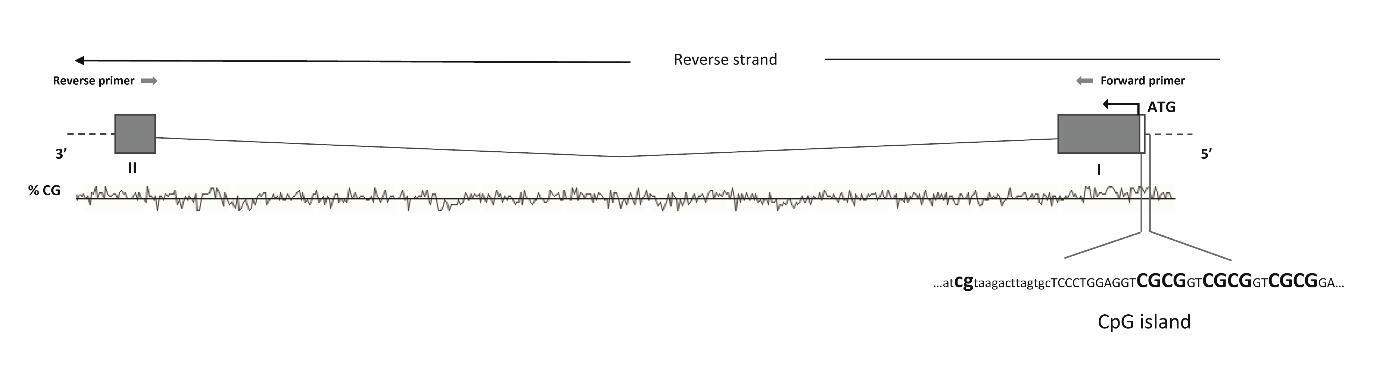


**Figure S1.** Schematic representation of rat *Oxtr* gene. Translation start codon (ATG), exons and introns are depicted. Coding regions of exons are shown darker. Sequence of CpG island studied is also reported. Bold text indicates the 7 CpG sites analyzed.


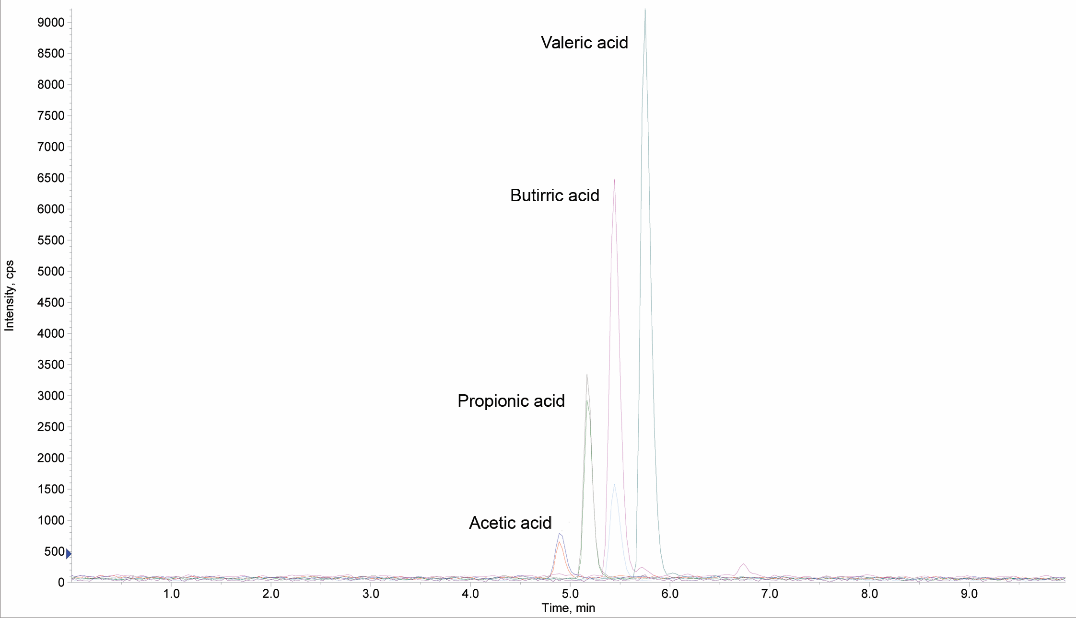


**Figure S2**. Chromatographic profile of the standard SCFA analytes at the concentration of 1 μg/mL.


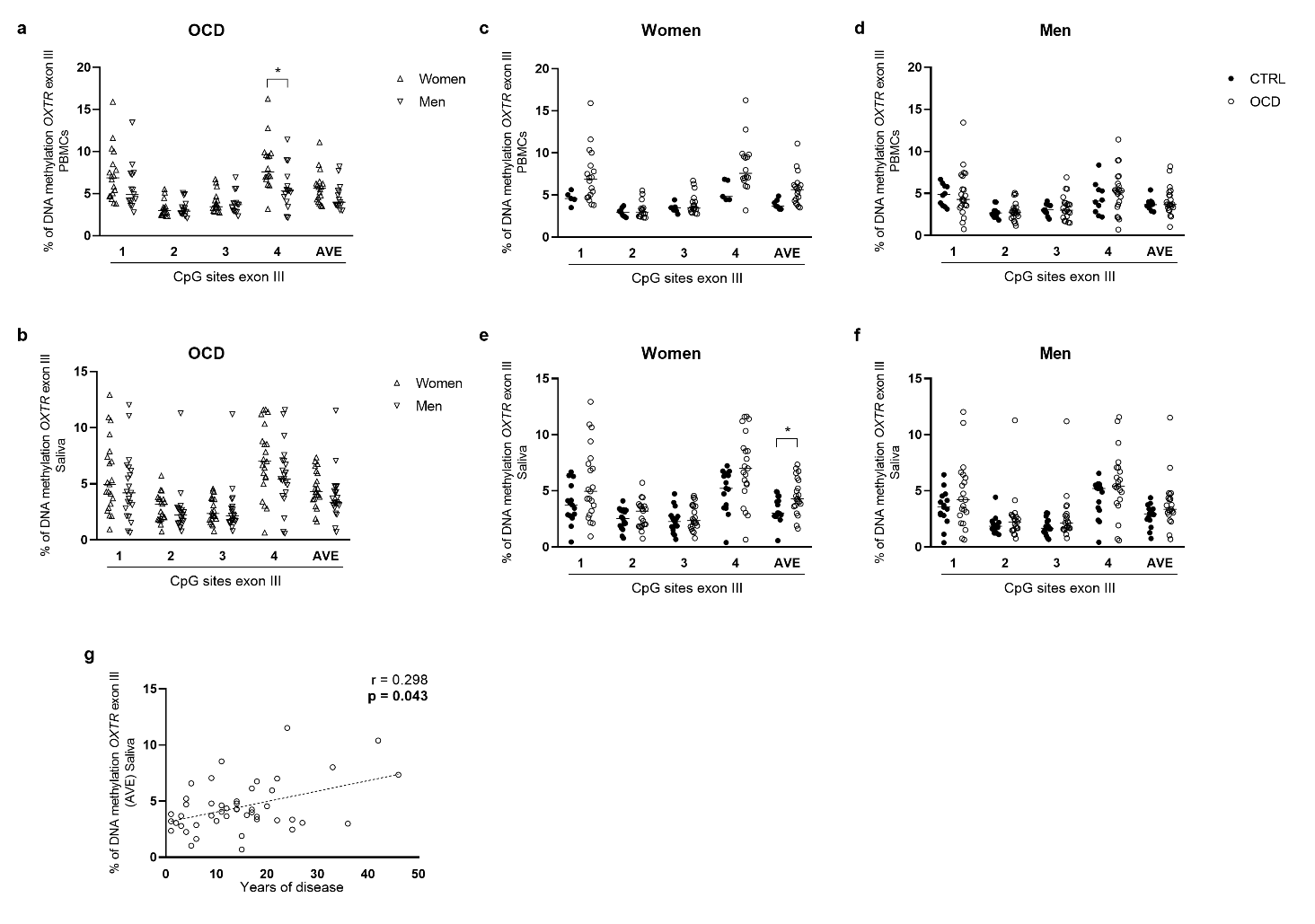


**Figure S3.** DNA methylation at *OXTR* exon III in human OCD PBMCs (**a**) and saliva (**b**) gender-stratified also compared to CTRLs again for PBMCs (c-d) and saliva (e-f). Scattered plots represent the mean of the % of methylation values of individual CpG sites under study as well as of the average (AVE) of the 4 CpG sites ± SEM. (**g**) correlation between % change of DNA methylation (AVE) in saliva of OCD subjects and years of disease. Data were compared by Spearman's rank correlation coefficient.


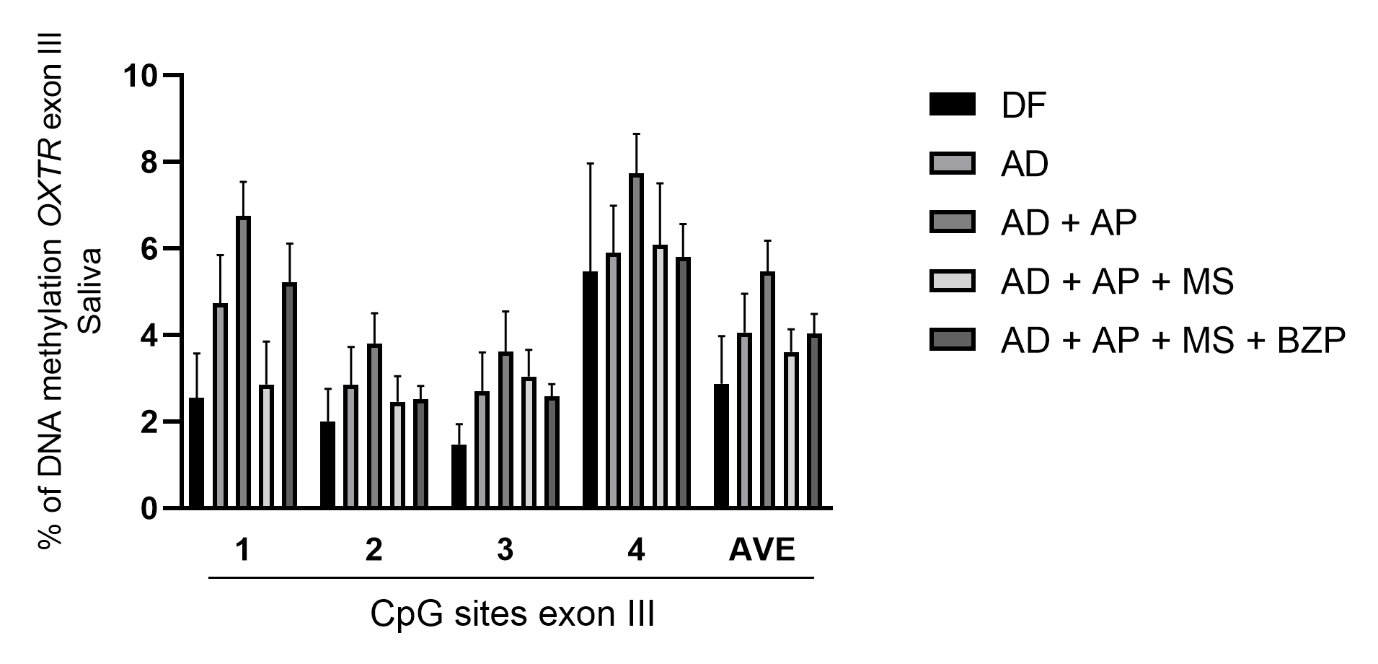


**Figure S4.** % of DNA methylation at *OXTR* exon III in human saliva samples of OCD subjects stratified based on drug therapy for the 4 CpG sites under study as well as their average (AVE). DF = drug free; AD = antidepressants; AP = antipsychotics; MS = mood stabilizers; BZP = benzodiazepine. Bonferroni multiple comparison test, adjusted P value > 0.999.


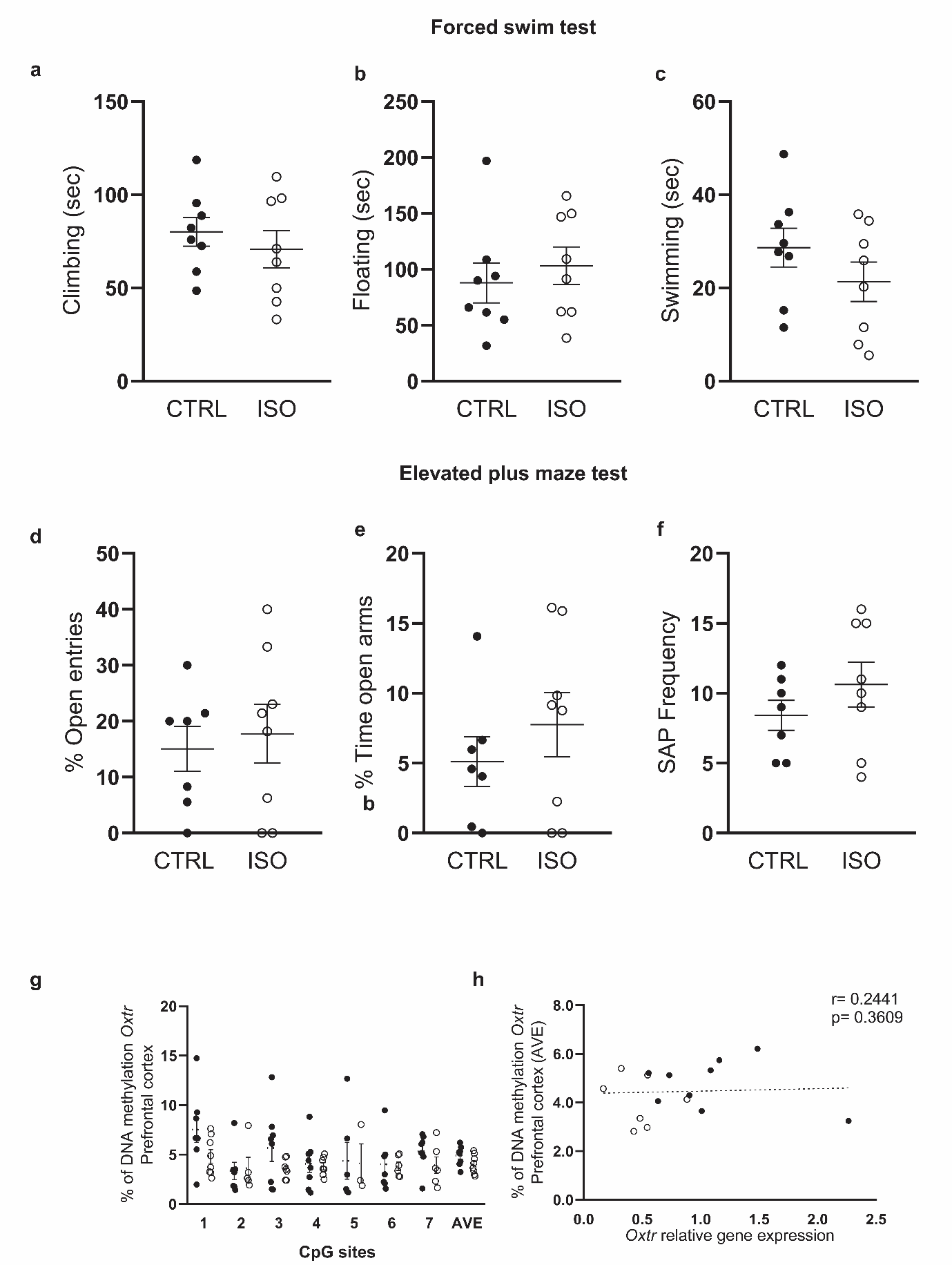


**Figure S5. (a-c)** *Effect of social isolation on forced swim test in Wistar male rats*. In the forced swim test ISO-groups showed no differences compared to CTRL animals in the parameters of time of climbing (a) time of floating (b) and time of swimming (c) at PND57 (*n* (CTRL=8, *n* ISO=8)). **(d-f)** *Effect of isolation on elevated plus maze test in Wistar male rats*. In the elevated plus maze rats ISO-groups no differences compared to CTRL animals in the parameters of percentage of time spent on the open arms (d), the percentage of open arm entries (e) and in the parameter of SAP frequency (f) at PND58 (*n* (CTRL=7, *n* ISO=8)).

**(g)** Comparison of *Oxtr* DNA methylation levels in rat PFC promoter between CTRL (empty circles) and ISO (filled circles) for the 7 CpG sites under study as well as for the average (AVE) of the 7 CpG sites. **(h)** Spearman correlation analysis between % of changes in *Oxtr* DNA methylation (AVE of the 7 CpG sites) and mRNA levels in rat PFC. Dashed line indicates Spearman’s r; the filled circles represent ISO rats while the empty circles represent CTRLs.

***References***

1. Melancia F, Schiavi S, Servadio M, Cartocci V, Campolongo P, Palmery M, et al. Sex-specific autistic endophenotypes induced by prenatal exposure to valproic acid involve anandamide signalling. Br J Pharmacol. 2018;175.

2. Servadio M, Melancia F, Manduca A, Di Masi A, Schiavi S, Cartocci V, et al. Targeting anandamide metabolism rescues core and associated autistic-like symptoms in rats prenatally exposed to valproic acid. Transl Psychiatry. 2016;6.

3. Yankelevitch-Yahav R, Franko M, Huly A, Doron R. The forced swim test as a model of depressive-like behavior. J Vis Exp. 2015;2015.

4. Livak KJ, Schmittgen TD. Analysis of relative gene expression data using real-time quantitative PCR and the 2-ΔΔCT method. Methods. 2001;25.

5. Caputi FF, Di Benedetto M, Carretta D, Bastias del Carmen Candia S, D’Addario C, Cavina C, et al. Dynorphin/KOP and nociceptin/NOP gene expression and epigenetic changes by cocaine in rat striatum and nucleus accumbens. Prog Neuro-Psychopharmacology Biol Psychiatry. 2014;49.

6. D’Addario C, Bellia F, Benatti B, Grancini B, Vismara M, Pucci M, et al. Exploring the role of BDNF DNA methylation and hydroxymethylation in patients with obsessive compulsive disorder. J Psychiatr Res. 2019;114.

7. Koliada A, Syzenko G, Moseiko V, Budovska L, Puchkov K, Perederiy V, et al. Association between body mass index and Firmicutes/Bacteroidetes ratio in an adult Ukrainian population. BMC Microbiol. 2017;17.

8. González-Hernández LA, Ruiz-Briseño MDR, Sánchez-Reyes K, Alvarez-Zavala M, Vega-Magaña N, López-Iñiguez A, et al. Alterations in bacterial communities, SCFA and biomarkers in an elderly HIV-positive and HIV-negative population in western Mexico. BMC Infect Dis. 2019;19.
